# Viability Under Degraded Control Authority

**DOI:** 10.1109/lcsys.2023.3342059

**Published:** 2023-12-12

**Authors:** Hamza El-Kebir, Richard Berlin, Joseph Bentsman, Melkior Ornik

**Affiliations:** Department of Aerospace Engineering, University of Illinois Urbana–Champaign, Urbana, IL 61801 USA; Department of Trauma Surgery, Carle Hospital, Urbana, IL 61801 USA; Department of Mechanical Science and Engineering, University of Illinois Urbana–Champaign, Urbana, IL 61801 USA.; Department of Mechanical Science and Engineering, University of Illinois Urbana–Champaign, Urbana, IL 61801 USA; Department of Aerospace Engineering and the Coordinated Science Laboratory, University of Illinois Urbana–Champaign, Urbana, IL 61801 USA

**Keywords:** Fault accommodation, fault detection, system identification

## Abstract

In this letter, we solve the problem of quantifying and mitigating control authority degradation in real time. Here, our target systems are controlled nonlinear affine-in-control evolution equations with finite control input and finite- or infinite-dimensional state. We consider two cases of control input degradation: finitely many affine maps acting on unknown disjoint subsets of the inputs and general Lipschitz continuous maps. These degradation modes are encountered in practice due to actuator wear and tear, hard locks on actuator ranges due to over-excitation, as well as more general changes in the control allocation dynamics. We derive sufficient conditions for identifiability of control authority degradation, and propose a novel real-time algorithm for identifying or approximating control degradation modes. We demonstrate our method on a nonlinear distributed parameter system, namely a one-dimensional heat equation with a velocity-controlled moveable heat source, motivated by autonomous energy-based surgery.

## I. Introduction

IN CONTROL systems, fault detection and mitigation is key in ensuring prolonged safe operation in safety-critical environments [1]. Any physical system undergoes gradual degradation during its operational life cycle, for instance due to interactions with the environment or from within as a result of actuator wear and tear. Gradual degradation or impairment, as the name suggests, often reduces the performance of a system in cases when potential degradation modes were not taken into account during control synthesis. Fault tolerance is a key property of systems that are capable of mitigating or withstanding system faults, including gradual degradation.

A number of stochastic approaches to fault identification and mitigation have been developed in the past, with the main objective of estimating the *remaining useful life* (RUL) of a system, and how this metric is influenced by the controller. Mo and Xie [2] developed an approach to approximate the loss in effectiveness cause by actuator component degradation using a reliability value. Their method relies on frequency domain analysis using the Laplace transform, which is limited to linear systems; in turn, proposed reliability improvements hinge on the use of a PID controller strategy and rely on a particle swarm optimization routine, which is highly restrictive with regard to runtime constraints and convergence guarantees. A similar approach was developed by Si et al. [3], where reliability was assessed using an event-based Monte Carlo simulation approach, wherein potential degradation modes are simulated *en masse*, further limiting the applicability of this method. This is due to the intractable number of potential failure modes that may be encountered in practice, which would demand a very large number of Monte Carlo simulations.

In the deterministic setting, Wang et al. [4] considered control input map degradation and actuator saturation in discrete-time linear systems, where a fault-tolerant control is developed by solving a constrained optimization problem. Given the discrete-time linear system setting, [4] uses efficient linear matrix inequality (LMI) techniques for controller synthesis. However, the class of actuator degradations considered in [4] is limited to linear diagonal control authority degradation with input saturation. In the context of switching systems, Niu et al. [5] considered the problem of active mode discrimination (AMD) with temporal logic-constrained switching, where a set of known switching modes was known *a priori*. Other work relies on application-specific projection-based adaptation routines where a set of switching adaptive controllers is used [6]; however, such an approach does not incorporate non-smooth failure modes that conditionally act depending on the control input provides, nor are regions of degradation robustly identified as is done in the present work. A major contribution is the fact that we do not demand complete knowledge of the underlying system structure, which is essential in Lyapunov analysis for adaptive methods.

**Fig. 1.**
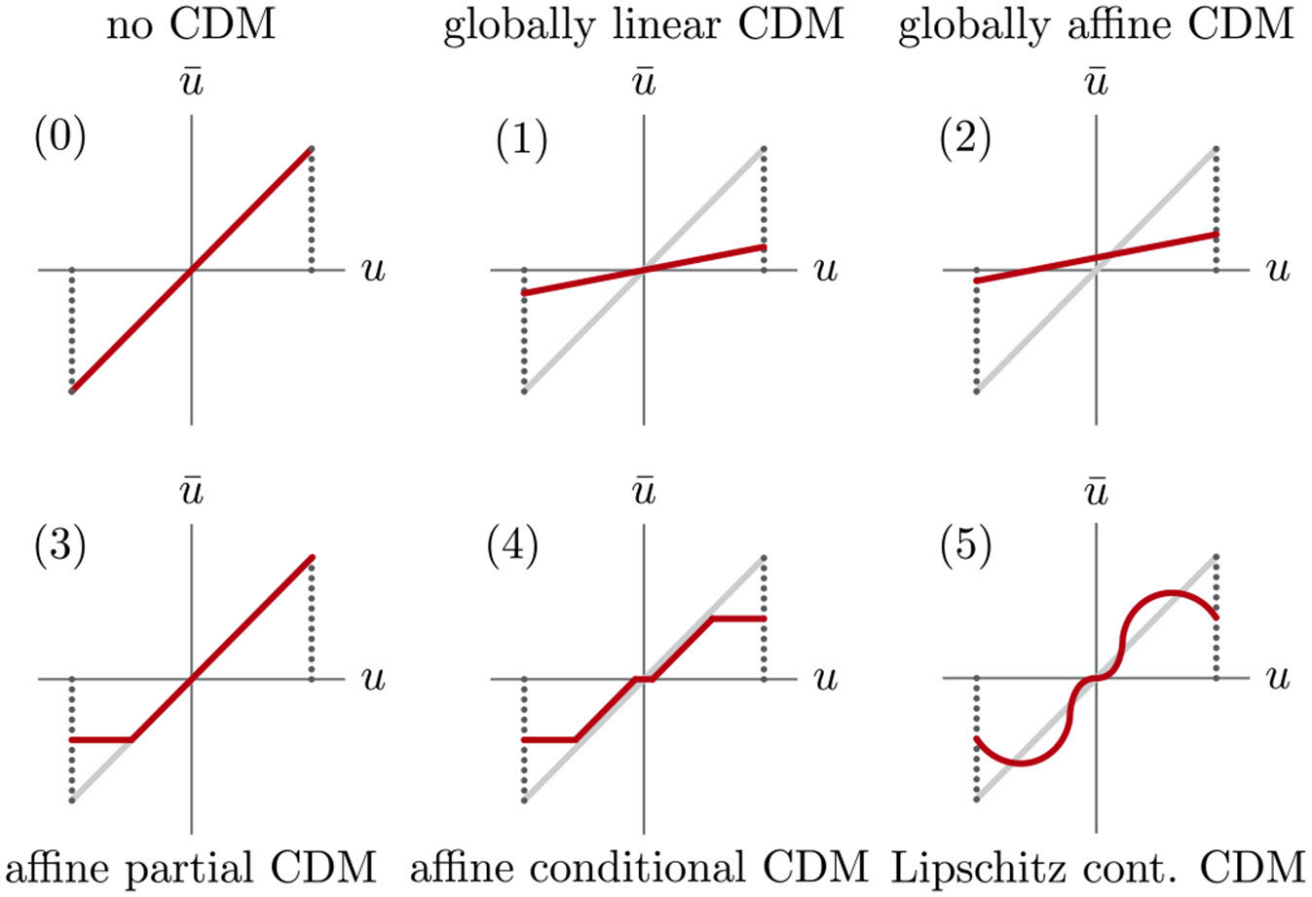
Comparison between various classes of control authority degradation maps.

In the present work, we consider a class of faults, which we refer to as *actuator degradation*. The latter may arise as a result of wear and tear, software errors, or even adversarial intervention. Considering the following nonlinear control-affine dynamics $\dot{x}(t)=f(x(t))+g(x(t))u(t)$, we define input degradation modes of the form $\dot{\bar{x}}(t)=f(\bar{x}(t))+Rg(\bar{x}(t))Pu(t)$, where P and R are two unknown time-varying maps. We refer to P as a *control authority degradation map* (CDM), whereas R is referred to as a *control effectiveness degradation map* (CEM). Our focus in this letter is on CDMs; a number of common CDMs are illustrated in Fig. 1. A CDM P effectively acts as a control input remapping, and can be thought of in the context of control systems with delegated control allocation, e.g., when an actuator with internal dynamics takes u(t) and remaps it based on its internal state. Such a setting includes common degradation modes such as deadzone or saturation, or any other nonlinear transformation due to effects such as friction. In more extreme cases, it is possible that P maps a control signal $u_{i}(t)$ to another control signal $u_{j}(t)$ due to incorrect wiring or software design. The types of control authority degradation maps that we allow for in this letter go beyond linear maps applied to discrete-time finite-dimensional linear systems, which hitherto been the main focus in prior work. We develop an *efficient passive algorithm for detection and identification of CDMs*, with the quality of the reconstructed CDM monotonically increasing with system run time. We have chosen to focus on the viability property in this letter as opposed to discussing *viabilizability*, i.e., synthesizing fault-tolerant controllers. Hence, the focus of this letter is on guaranteed identification of control authority degradation modes, where sufficient conditions on identifiability and convergence results are developed.

We note that we do not consider external disturbances or other unmodeled dynamics in this letter; robustness results regarding the effects of disturbances will be the subject of future work. A more general analysis capturing faults beyond CDMs will also be deferred to a forthcoming publication; instead, we have elected to give in-depth convergence results. The results of this letter allow for *guaranteed approximation of arbitrary control degradation maps* without the need for knowledge of possible degradation modes or handcrafted filters, addressing an open problem in the literature The natural next step of this letter, outside of the scope of this letter, is to approximate unviable control signal with their closest viable counterpart, with robustness bounds on the maximum trajectory deviation.

## II. Preliminaries

### A. Notation

We use ‖⋅‖ to denote the Euclidean norm. Given two sets A, $B\;\subseteq \;R^{n}$, we denote by A+B their Minkowski sum {a+b:a∈A,b∈B}; the Minkowski difference is defined similarly. By 2^*A*^ we refer to the power set of A, i.e., the family of all subsets of A. We denote a closed ball centered around the origin with radius r>0 as ${ℬ}_{r}$. By ℬ(x,r) we denote $\{x\}+{ℬ}_{r}$. We denote by ℒ(A,B) the set of bounded linear operators, and by 𝒞(A,B) the set of closed linear operators between A and B. We define $R_{+}≔[0,\infty )$. For two points in a Banach space ℬ∋a,b let [a,b] denote the convex hull of a and b, i.e., [a,b]≔conv{a,b}. Given a point x∈S and a set A⊆S, we denote $d(x,A)\;≔{\text{inf}}_{y\in A}\;d(x,y)$. We define the distance between two sets $A,B\subseteq R^{n}$ to be $d(A,B)\;≔{\text{sup}}_{a\in A}\;{\text{inf}}_{b\in B}\|a-b\|$. We denote the Hausdorff distance as $d_{H}(A,B)\;≔\max\{d(A,B),d(B,A)\}$. An alternative characterization of the Hausdorff distance reads $d_{H}(A,B)=\text{inf}\{\rho \geq 0\;:A\subseteq B_{+\rho },B\subseteq A_{+\rho }\}$, where $X_{+\rho }$ denotes the *ρ-fattening* of X, i.e., $X_{+\rho }≔{⋃}_{x\in X}\{y\in R^{n}\;:\|x-y\|\leq \rho \}$.

We denote by ∂A the boundary of A in the topology induced by the Euclidean norm. For a function g:A→B, we denote by $g^{-1}$ the inverse of this function if an inverse exists and otherwise denoting the preimage. By dom(*g*) we refer to the domain of the function (in this case A). We denote by $g^{†}$ the Moore–Penrose pseudo-inverse of a linear function g. We use the Iverson bracket notation ⟦⋅⟧, where the value is 1 if the expression between the brackets is true, and 0 otherwise.

In this letter, we shall consider star-shaped sets, which are defined as follows:

*Definition 1 (Star-Shaped Set and MGFs):* We call a closed compact set K⊆ℬ *star-shaped* if there exist (i) ς∈K, and (ii) a unique function $\varrho \;:{ℬ}_{1}\to R_{+}$, such that: $K={⋃}_{l\in {ℬ}_{1}}[\varsigma ,\varsigma +\varrho (l)l]$ where ${ℬ}_{1}$ denotes the unit ball in ℬ. We call ϱ a *Minkowski gauge function* (MGF), and ς the *star center*.

### B. Problem Formulation

Consider a known nonlinear control-affine system of the form of

(1) $$\dot{x}(t)=f(x(t))+g(x(t))u(t),$$

where x∈X, u∈U⊆𝒰, X and 𝒰 are Hilbert spaces, and f:X→X and g:X→ℒ(𝒰,X). In this letter, we assume $𝒰=R^{m}$. In addition, we assume that U is a *star-shaped* subset of $R^{m}$ such that span $U=R^{m}$. Finally, we assume that the full-state of the degraded system,

(2) $$\dot{\bar{x}}(t)=f(\bar{x}(t))+Rg(\bar{x}(t))Pu(t),$$

is known without error.

In system (2), a control action degradation map R can model changes in the control allocation function g, which may include actuator reconfiguration, such as a change in the trim angle on aircraft control surfaces, or misalignment of actuators due to manufacturing imperfections or wear and tear. Since R acts after g, it does not directly remap the control signal u(t), but it changes the action of a control input on the system; we therefore talk about control effectiveness, as opposed to control authority in the case of P, which acts before g. Changes in the drift dynamics f(x(t)) will not be treated in this letter.

In addition to identifying or approximating CDM P, we are interested in ‘undoing’ the effects of control authority degradation as much as possible. In particular, we are interested in the set of control signals (1) that can still be replicated in (2) when the CDM is acting; we call this the set of *viable control inputs*, $U_{v}$. With knowledge of P, we develop in this letter a method to obtain, for $u_{\text{cmd}}\in U_{v}$, $u_{v}$ such that ${Pu}_{v}$; here, $u_{\text{cmd}}$ and $u_{v}$ are called commanded and viabilized control inputs, respectively. This approach is closely related to a technique known in the literature as *fault hiding* [7]. Fault hiding is achieved by introducing an output observer based on the output of the degraded system, and augmenting the nominal system model by introducing so-called virtual actuators, which requires a nonlinear reconfiguration block that is strongly dependent on the underlying problem structure and failure modes [7, Sec. 3.6, p. 42]. In the setting considered in this letter, we show that we can adopt the fault hiding philosophy under much less stringent constraints for a general class of systems and degradation modes.

In this letter, we are interested in modeling unknown degraded system dynamics (2) for a time-invariant control authority degradation map (CDM) $P\;:U\;\to \;\bar{U}$, and no control effectiveness degradation (i.e., R=I). This amounts to reconstructing, or *identifying*, P.

*Problem 1 (Identifiability of Control Authority Degradation Maps):* For a class of time-invariant CDMs 𝒫∈P, if possible, identify P based on a finite number of full state, velocity, and control input observations $\left(\bar{x}(t),\;\dot{\bar{x}}(t),\;u(t)\right)$ of the degraded system.

Ideally, we would like to identify general nonlinear CDMs with known bounds on the approximation error. We illustrate the control authority degradation modes that are covered in this letter in Fig. 1.

We now proceed by solving Problem 1 for an unknown multi-mode affine CDMs, which allows for approximating Lipschitz continuous nonlinear CDMs with bounded error.

## III. Identifiability of Control Authority Degradation Maps

We now consider Problem 1. Let us assume that for U, the Minkowski gauge function ϱ is known. Let $P\;:U\;\to \;\bar{U}$ be an unknown control authority degradation map (CDM). We assume that $\bar{U}$ is also a star-shaped set, providing conditions on P and U under which this holds. It bears mentioning that star-shaped sets are more general than convex sets; most results presented in this letter will apply to star-shaped sets, which include polytopes, polynomial zonotopes, and ellipsoids.

Before we provide any results on the identifiability of control authority degradation modes, we pose the following key assumption on the nominal system dynamics (1). We allow for an *infinite-dimensional* state-space X, that is to say, X is a set of *functions*, but $X=R^{n}$ is also captured:

*Assumption 1:* For system (2), assume that

1. g(x) has closed range for all x∈X;
2. g(x) is injective for all x∈X, i.e., ker(g(x))={0};
3. $\dot{x}$ is known at some x∈X with u=0.

*Remark 1:* In the case of finite-dimensional systems, i.e., $X\subseteq R^{n}$, the first two conditions of Assumption 1 can be stated as:

1. The system is not overactuated, i.e., m≤n;
2. g(x) is of full-column rank for all x∈X.

We shall consider the case of multiple control degradation modes acting throughout the space U. The simplest of the so-called *conditional control authority degradation modes* (c-CDMs) acts only on a compact subset of U; we refer to these c-CDMs as *partial control authority degradation modes* (p-CDMs). Consider two compact star-shaped sets $\overset{ˇ}{U},\hat{U}\subseteq U$, and two p-CDMs $P_{\overset{ˇ}{U}}(u)≔u+⟦u\in \overset{ˇ}{U}⟧(P-I)u$, $P_{\hat{U}}(u)≔u+⟦u\notin \hat{U}⟧(P-I)\;u$, for some control degradation map P. Here, $P_{\overset{ˇ}{U}}$ is an *internally acting* partial CDM (i.e., acting inside $\overset{ˇ}{U}$), whereas $P_{\hat{U}}$ is an *externally acting* partial CDM (acting outside $\hat{U}$); when this distinction is immaterial, we use a combined hat and check symbol (e.g., U^ˇ), where U^ˇ is simply called the *affected set* of control inputs.

In reconstructing an *N*-mode c-CDM, we face the problem of discerning which control inputs belong to which conditional degradation mode. To make this problem tractable, we pose the following assumption:

*Assumption 2:* Let the internally acting *N*-mode c-CDM satisfy the following properties:

1. The number of modes N is known;
2. $\overset{ˇ}{𝒰}$ is a family of convex sets;
3. $\overset{ˇ}{𝒫}$ is a family of affine maps denoted by $Q_{i}=p_{i}+P_{i}$.
4. There exists a known δ>0, such that for all i≠j, $d_{H}(({\overset{ˇ}{U}}_{i},{\overset{ˇ}{P}}_{i}{\overset{ˇ}{U}}_{i}),({\overset{ˇ}{U}}_{j},{\overset{ˇ}{P}}_{j}{\overset{ˇ}{U}}_{j}))\geq \delta$.

**Fig. 2.**
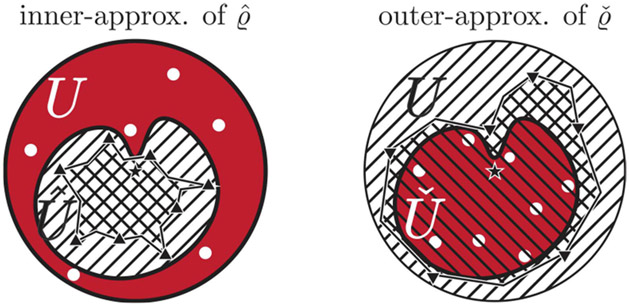
Comparison between inner- and outer-approximations of $\hat{U}$ and $\overset{ˇ}{U}$ respectively, based on Proposition 1 and Theorem 1 for a 1-mode c-CDM. The region with top-right-pointing hatching indicates the set in which the control input is unaffected; the red-colored region indicates the affected set. The respective approximations of U^ˇ allow one to find regions in which control inputs are guaranteed to be unaffected. In the left image, the set indicated by top-left-pointing hatching is an inner-approximation of $\hat{U}$, and in the right image this set is an outer-approximation of $\overset{ˇ}{U}$.

Assumption 2.4) ensures that the graph of any conditional CDM is sufficiently distinct from any other CDM to allow for distinguishability. We are also interested in obtaining outer-approximations of $\overset{ˇ}{U}$ and inner-approximations of $\hat{U}$ for each degradation mode, as illustrated in Fig. 2, so that we can restrict control inputs to regions that are guaranteed to be unaffected. Since we only have access to a finite number of control input samples, we pose the following assumption regarding the regularity of the MGF associated with PU^ˇ.

*Assumption 3:* Assume that U^ˇ has star center ςˇ^=0, and assume that the MGF ϱˇ^ associated with U^ˇ is Lipschitz continuous, i.e., there exists a known Lˇ^ such that ∣ϱˇ^(l)−ϱˇ^(l′)∣≤Lˇ^‖l−l′‖, for all $l,l^{\prime }\in {ℬ}_{1}$.

We now proceed to show that Assumption 3 holds for the image of Lipschitz star-shaped sets under affine maps.

*Lemma 1:* Given a star-shaped set U characterized by a Lipschitz MGF ϱ and star center ς, the range of U under an affine map Qu≔p+Pu is also a star-shaped set with Lipschitz MGF.

We can now pose a key result on the guaranteed approximation of Lipschitz MGFs from a finite set of samples.

*Proposition 1:* Assume that Assumption 3 holds for the unknown MGFs $\overset{ˇ}{\varrho }$ and $\hat{\varrho }$. Then, for some given $\overset{ˇ}{u}$, ${\overset{ˇ}{u}}^{\prime }\in \overset{ˇ}{U}$ and $\hat{u}$, ${\hat{u}}^{\prime }\notin \hat{U}$, we have for all μ∈[0,1]:

(3) $$\overset{ˇ}{\varrho }\left(\frac{\mu \overset{ˇ}{l}+(1-\mu ){\overset{ˇ}{l}}^{\prime }}{\left\|\mu \overset{ˇ}{l}+(1-\mu ){\overset{ˇ}{l}}^{\prime }\right\|}\right)\;\;\leq \text{min}\left\{\left\|\overset{ˇ}{u}\|+(1-\mu )\overset{ˇ}{L}\|l-l^{\prime }\|,\|{\overset{ˇ}{u}}^{\prime }\|+\mu \overset{ˇ}{L}\|\overset{ˇ}{l}-{\overset{ˇ}{l}}^{\prime }\right\|\right\},$$

and

(4) $$\hat{\varrho }\left(\frac{\mu \hat{l}+(1-\mu ){\hat{l}}^{\prime }}{\left\|\mu \hat{l}+(1-\mu ){\hat{l}}^{\prime }\right\|}\right)\;\;\geq \text{max}\left\{0,\;\|\hat{u}\|+(1-\mu )\hat{L}\|\hat{l}-{\hat{l}}^{\prime }\|,\|{\hat{u}}^{\prime }\|-\mu \hat{L}\|\hat{l}-{\hat{l}}^{\prime }\|\right\},$$

where lˇ^:=u^ˇ∕‖u^ˇ‖ and lˇ^′:=u^ˇ′∕‖u^ˇ′‖.

*Proof:* This result follows directly from non-negativity of the MGF and the mean value theorem, given the Lipschitz continuity of ϱˇ^ as assumed in Assumption 3. ■

The results given in Proposition 1 allow for direct inner-approximation of $\hat{U}$ and outer-approximation of $\overset{ˇ}{U}$ through guaranteed interpolation; these results will allow us to restrict closed-loop control inputs to a subset of U that is *guaranteed to be unaffected by P* as illustrated in provided in Fig. 2. The method for approximating U^ˇ will be rigorized in the next theorem.

We now pose the main result on the identifiability of *N*-mode conditional control authority degradation modes (c-CDMs), where multiple affine CDMs act on disjoint subsets of U; this will allow us to approximate of Lipschitz continuous CDMs as shown at the end of the next section.

*Theorem 1 (Reconstructing N-mode Affine c-CDMs):* Consider system (2) and Assumptions 1-2. Assume that the c-CDM is represented by *N* unknown internally acting *affine* maps $Q_{i}$, each acting on mutually disjoint unknown star-shaped sets ${\overset{ˇ}{U}}_{i}\subseteq U$, giving $Q_{\overset{ˇ}{𝒰}}$ as the p-CDM. Let there be a given array of distinct state–input pairs ${\left[(\bar{x}[i],u[i])\right]}_{i=1}^{N}$, and a corresponding array of degraded velocities ${\left[\dot{\bar{x}}[i]\right]}_{i=1}^{N^{\prime }}$ obtained from system (2), with $N^{\prime }\geq N(m+1)$. Let there also be a given array of undegraded state–input pairs ${\left[(x_{*}[i],u_{*}[i])\right]}_{i=1}^{M}$, with M≥m. Assume that there exist m state–input pairs indexed by J and $J_{*}$, such that the arrays of input vectors ${\left\{u[J_{j}]\right\}}_{j=1}^{m}$ and ${\left[u_{*}[J_{*},j]\right]}_{j=1}^{m}$ are linearly independent.

The CDM $Q_{\overset{ˇ}{𝒰}}$ can then be approximated as follows:

(5) Q~𝒰ˇu={uu∉Uˇouter,∑i=1N⟦u∈Uˇi,inner⟧Qiuu∈Uˇinner,inconclusiveu∈Uˇouter∖Uˇinner,}

*Proof:* We cluster the array [(u[i],u^ˇ[i])]i=1N′ into Nclusters with a Hausdorff distance of at least δ between each pair of clusters. If each cluster i contains at least m vectors u[i] that are linearly independent, then (5) can be obtained. Here, ${\overset{ˇ}{U}}_{\text{inner}}≔{⋃}_{i=1}^{N}{\overset{ˇ}{U}}_{i,\text{inner}}$ and ${\overset{ˇ}{U}}_{\text{outer}}≔{⋃}_{i=1}^{N}{\overset{ˇ}{U}}_{i,\text{outer}}$. Each $Q_{i}$ is obtained by considering for each cluster $\nu_{i}≔g^{†}(\bar{x}[j])(\dot{\bar{x}}[j]-f(\bar{x}[j]))$ where index j is not part of the array of linearly independent inputs indexed by j, $u≔[\;u[j_{1}]-\nu_{i}\;\cdots \;u[j_{m}]-\nu_{i}\;]$ and $\Delta u≔{\left[g^{†}(\bar{x}[j_{j}])[\dot{\bar{x}}[j_{j}]-f(\bar{x}[j_{j}]))-u[j_{j}]-\nu_{i}\right]}_{j=1}^{m}$. Linear operator $P_{i}$ is obtained as

(6) $$P_{i}=(u+\Delta u)u^{T}{\left({\mathrm{uu}}^{T}\right)}^{-1}.$$

The translation $p_{i}$ is obtained as $p_{i}=\nu_{j}-P_{i}u[j]$, which yields the *i*’th mode affine CDM $Q_{i}$:

(7) $$Q_{i}u≔p_{i}+P_{i}u.$$

Here, each affected set is approximated as follows: In case U^ˇi is internally acting (i.e., Uˇi=U^ˇi), (3) yields an outer-approximation to ${\overset{ˇ}{\varrho }}_{i}$ by taking a convex combination of the m basis vectors {lˇ^∗[j∗,j]=u∗[j∗,j]∕‖u∗[j∗,j]‖}j=1m and their values. Similarly, for externally acting U^ˇi (i.e., U^i=U^ˇi), (4) yields an inner-approximation to ${\hat{\varrho }}_{i}$ using m basis vectors {lˇ^[jj]}j=1m. Inner- and outer-approximations satisfy the relation U^ˇi,inner⊆U^ˇi⊆U^ˇi,outer (cf. Fig. 2).

We first consider a globally acting affine CDM. We obtain the closed-form expression of $P_{i}$, (6), by solving the quadratic program ${\text{min}}_{P\in ℒ(U,\bar{U})}{\left\|Pu-(u+\Delta u)\right\|}^{2}$, which yields a unique linear map P that maps u to u+Δu as desired. The translation term $p_{i}$ can be verified by direct substitution in (7), yielding the affine map $Q_{i}$.

In (6), since the inverse of $u^{T}u$ must be taken, we require both that u is a square matrix, and $u^{T}u$ is invertible. This is achieved by considering $u\in R^{m\times m}$ of full column rank, as guaranteed by the linear independence hypothesis.

Regarding $g^{†}(x)$, the Moore–Penrose pseudo-inverse is defined for a general Hilbert space X, provided that range(g(x)) is closed for all x∈X [8, Sec. 4.2, p. 47]. For $g^{†}(x)$ to be a left-inverse, a necessary condition is that g(x) be injective, i.e., ker(g(x))={0} for all x∈X [8, Cor. 2.13, p. 36]. Finally, the translation term p is accounted for as well in (7).

To approximate the *i*’th affected set, U^ˇi, we require a spanning set of basis vectors that lie within U^ˇi, as provided for in the hypotheses. The unknown MGF associated with U^ˇi can be obtained according to Proposition 1 using (3)-(4), where an inner-approximation is desired for internally acting p-CDMs, and outer-approximations for externally acting p-CDMs. These approximations are obtained through repeated convex combinations and the corresponding inequality given in (3)-(4), for a total of m times; an explicit expansion of the resulting expression is omitted here for the sake of space. ■

*Remark 2:* This result incorporates p-CDMs that map a set $\overset{ˇ}{U}$ to a constant, e.g., $Q_{\overset{ˇ}{𝒰}}\overset{ˇ}{U}\;=\;p$. To highlight the utility of this result, it should be noted that the *hypotheses given here allow for commonly encountered degradation modes such as deadzones and saturation to be modeled* (see Fig. 1(4)). Additionally, Theorem 1 allows for *discontinuous* control authority degradation modes, a property that is rarely present in prior work.

We can now consider the case in which P is a Lipschitz continuous CDM. We consider an approximation of P by an *N*-mode affine c-CDM $\tilde{P}$, for which we derive an explicit error bound given that the Lipschitz constant of P, $L_{P}$, is known.

*Theorem 2 (Approximating Lipschitz Continuous CDMs by N-Mode Affine c-CDMs):* Let the hypotheses of Theorem 1 hold, with the exception that $P≔Q_{\overset{ˇ}{𝒰}}$ is now an $L_{P}$-Lipschitz continuous CDM and Assumption 2 is now dropped. If N clusters that satisfy the linear independence requirements of Theorem 1 are identified, then the resulting *N*-mode affine c-CDM approximation $\tilde{P}$ has the following error:

For all $u\in {\overset{ˇ}{U}}_{i,\text{inner}}$ and all i=1,…,N,

(8) $$\|Pu-\tilde{P}u\|\leq \|\underset{j=1,\ldots ,m}{\text{min}}\varepsilon_{i,j}+L_{P}\|u[i,j]-u\|,$$

where $\varepsilon_{i,j}≔\|{Pu}_{i}[j]-{\overset{ˇ}{P}}_{i}u_{i}[j]\|$, and $u[i,j]≔u_{i}[j]$, where $u_{i}$ is an array composed of all control inputs in the *i*’th cluster.

*Proof:* The proof is similar to that of Theorem 1, with the error bound (8) following an application of the triangle inequality in combination with the Lipschitz continuity of P, the properties of the affine maps ${\overset{ˇ}{P}}_{i}$, and the known samples of (u,Pu). ■

We can now pose a convergence result on the *N*-mode affine c-CDM approximation $\tilde{P}$ of a Lipschitz continuous CDM P.

*Corollary 1:* Error bound (8) is monotonically decreasing in the number of samples $N^{\prime }$ and the number of c-CDM modes N. In the limit of the $N^{\prime },N\to \infty$, error bound (8) converges to zero.

*Proof:* In (8), $\varepsilon_{i,j}$ monotonically converges to zero, because the operator norm $\left\|P-{\overset{ˇ}{P}}_{i}\right\|$ restricted to the *i*’th cluster converges monotonically to zero; this fact follows by considering that the diameter of each cluster converges to zero for a greater number of samples and clusters, similarly to the proof of Lemma 2, as well as the fact that P is Lipschitz continuous, meaning that the total variation of P on this restriction decreases monotonically as well. Another consequence of the diminishing cluster diameter is that ‖u[i,j]−u‖ converges monotonically to zero. ■

**Fig. 3.**
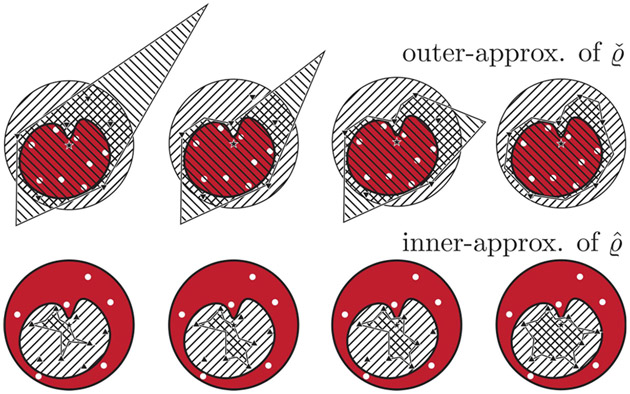
Comparison between inner- and outer-approximations of $\hat{U}$ and $\overset{ˇ}{U}$ respectively, based on Proposition 1 and Theorem 1 for an increasing number of samples for a 1-mode c-CDM. Clearly, for a larger number of points of sufficiently dispersed points, increasingly tight approximations are obtained as formalized in Lemma 2.

In the results given above, we find that it is in general impossible to uniquely determine each U^ˇ from finitely many samples. Intuitively, given a greater number of distinct points inside U^ˇ and U∖U^ˇ, it should be possible to more tightly approximate U^ˇ. This idea is illustrated in Fig. 3. We now state a lemma on the convergence of inner- and outer-approximations of the affected set U^ˇ.

*Lemma 2:* Consider ϵ>0, such that a given set of $N_{\epsilon }\geq m$ distinct pairs ((u,PU^ˇu)) denoted by ${𝒢U}_{N,\epsilon }$, satisfies Assumptions 1-3, where PU^ˇ is (i) an *N*-mode affine c-CDM, or (ii) a Lipschitz continuous CDM. Let ${𝒢U}_{N}$ be such that for each $u_{i}$ in ${𝒢U}_{N,\epsilon }$, ⋃i=1Nϵℬϵ(ui)⊇U^ˇ; i.e., ϵ-balls centered at each sampled control input form a cover of U^ˇ. Let U^ˇinnerNϵ and U^ˇouterNϵ denote the corresponding inner- and outer-approximations of U^ˇ using the procedure given in Theorem 1 from ${𝒢U}_{N,\epsilon }$. Then, we have U^ˇinnerNϵ⊆U^ˇinnerNϵ′⊆U^ˇ and U^ˇ⊆U^ˇouterNϵ′⊆U^ˇouterNϵ for all $\epsilon^{\prime }\;<\;\epsilon$. In addition, we have limϵ→0U^ˇinnerNϵ=limϵ→0U^ˇouterNϵ=U^ˇ.

*Proof:* Since it is assumed that the pairs in ${𝒢U}_{N,\epsilon }$ are distinct, the approximations of $\overset{ˇ}{\varrho }$ and $\hat{\varrho }$ obtained in Theorem 1 will become increasingly tight for decreasing ϵ, since the expressions derived in Theorem 1 will rely increasingly less on the Lipschitz bound assumption. Since dH(U^ˇinnerNϵ,U^ˇouterNϵ) is monotonically decreasing for decreasing ϵ, in the limit of ϵ→0, both sequences will converge to U^ˇ in the Hausdorff distance. This follows from the fact that the Hausdorff distance between the boundary of U^ˇ and the sampled points u decreases monotonically with decreasing ϵ, leading to tighter approximations of $\hat{\varrho }$ and $\overset{ˇ}{\varrho }$ as per Proposition 1. ■

## IV. Application

We consider an infinite-dimensional system based on a 3D model of tissue thermodynamics during electrosurgery [9]:

(9) $$\dot{z}(t,\xi )=a\nabla^{2}z(t,\xi )+q(\xi )u_{1},\;\;\dot{d}(t)=z(t,1)\;u_{2}$$

where u∈[0,10]×[0,1]. The unit heat source is modeled as $q(\xi )=\frac{1}{\epsilon }⟦\xi \in [0,\epsilon ]⟧$, for some known ϵ>0. This model approximates a slab of tissue with the state representing the surface temperature; $u_{1}$ denotes the input power and $u_{2}$ denotes the needle depth.

For simplicity, we set the input power $u_{1}=1$, and consider only the needle depth $u_{2}$ as the free control input. We can express system (9) as affected by a CDM P as:

$$\dot{x}(t,\xi )=\left[\begin{array}{c} \dot{z}(t,\xi )\\ \dot{d}(t) \end{array}\right]=\left[\begin{array}{c} {a\nabla }^{2}x_{1}(t,\xi )\\ 0 \end{array}\right]+\left[\begin{array}{cc} q(\xi ) & 0\\ 0 & 1 \end{array}\right]P\left[\begin{array}{c} u_{1}\\ u_{2} \end{array}\right].$$

**Fig. 4.**
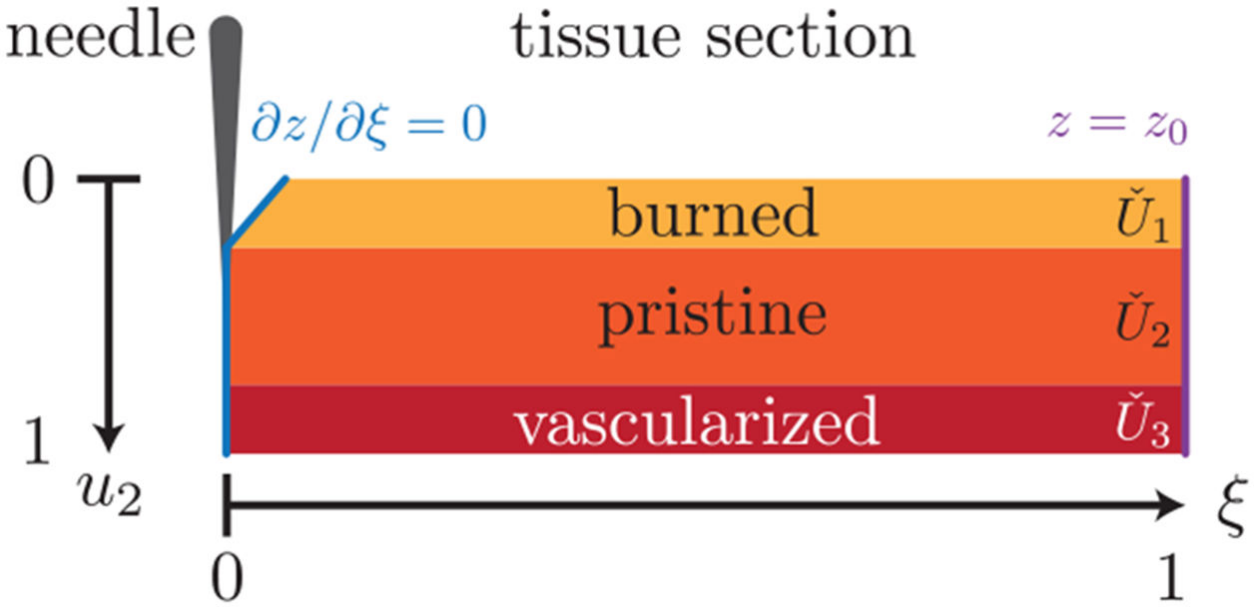
Illustration of the domain on which (9) acts. There are three regions of degradation, of which the burned and the vascularized region form two; the last region is not shown, but saturates the $u_{1}$ to 5.

**Fig. 5.**
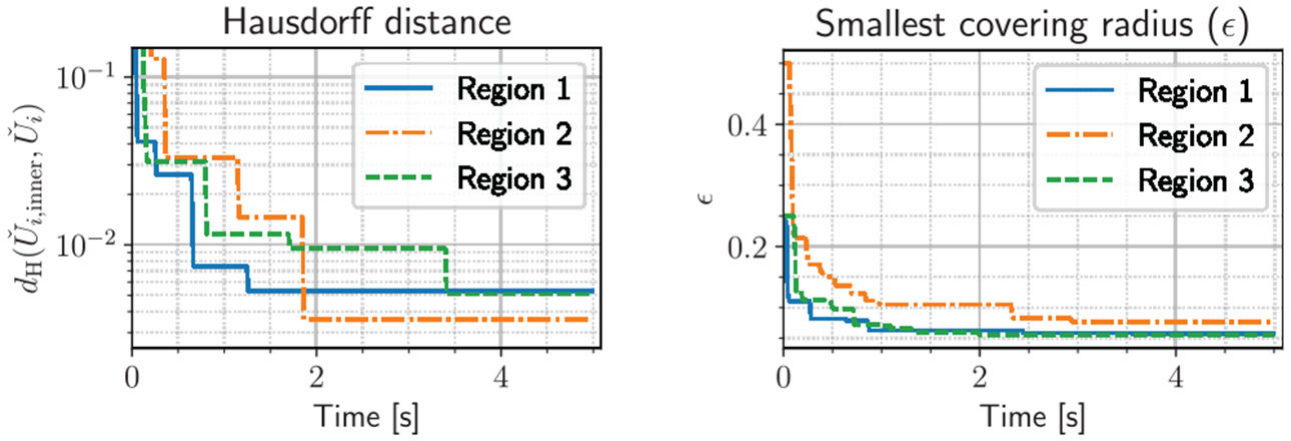
Hausdorff distance error for the inner approximations (left) and minimal covering radius (right) of the three affected regions on which the c-CDM of (9) acts as a function of time.

We consider a CDM of the form $Pu=\left[\begin{array}{cc} I & P_{12}\\ 0 & I \end{array}\right]u$, where $P_{12}$ is the map to be identified. We are interested in a 3-mode piecewise linear CDM $P_{12}$, with ${\overset{ˇ}{U}}_{1}\;=\;[0,0.25]$, ${\overset{ˇ}{U}}_{2}\;=\;[0.5,0.75]$, and ${\overset{ˇ}{U}}_{3}\;=\;[0.75,1]$; these regions are illustrated in Fig. 4. Region 1 corresponds to a charred region at the top of the tissue where the needle does not fully contact the tissue. Region 2 is a layer of pristine tissue, where the original dynamics act. Region 3 is a layer of highly vascularized tissue, in which a large fraction of heat that is added to the system gets transported away. We consider a piecewise linear function $P_{12}p≔(0.25+3p)⟦p\;<\;0.25⟧+⟦0.25\;\leq \;p\;\leq \;0.75⟧+(2.5-2p)⟦p>0.75⟧$. We consider a sinusoidal control signal for the probe depth with a period of 0.3 seconds, $u_{2}(t)=(1-\cos(20\pi t∕3))∕2$, and a state–input sampling frequency of 20 Hz. We assume stochastic sampling periods, where the time is perturbed with a uniform 0.01 second error to model signal processing delays. The underlying goal of this application is to perform passive probing of the affected tissue layers and reconfigure the thermodynamics model to account for tissue damage, as is commonly encountered in electrosurgery. Fig. 5 shows on the left the Hausdorff distance between each affected region over time to show that approximations become tighter with time, according to the decreasing minimal covering radius ϵ (right), as shown in Lemma 2. After three samples in each region, we uniquely identify the appropriate affine map, but the inner-approximation of the affected region is refined passively over time.

## V. Conclusion

In this letter, we have introduced the concept of a *control authority degradation map* (CDM). We have proved conditions on the identifiability of a broad class CDMs, including *N*-mode affine CDMs and Lipschitz continuous CDMs, for a class of affine-in-control nonlinear systems. Based on the identifiability results, we have formulated a constructive method for reconstruction or approximating CDMs, with explicit bounds on the approximation error. Our CDM identification method is executable in real time, and is guaranteed to monotonically decrease in error as more full-state observations become available. In future work, we aim to incorporate stochastic process and output noise into the presented framework.
